# Micro- and Mesoplastic Consumption Tendency of *Exaiptasia diaphana* Sea Anemones

**DOI:** 10.3390/ani15030405

**Published:** 2025-02-01

**Authors:** Anita Kaliszewicz, Agata Czyżewska, Kamil Karaban, Izabella Olejniczak, Paweł Boniecki

**Affiliations:** 1 Institute of Biological Sciences, Cardinal Stefan Wyszynski University in Warsaw, Wóycickiego 1/3, 01-938 Warsaw, Poland; k.karaban@uksw.edu.pl (K.K.); iza-olejniczak@wp.pl (I.O.); pawbon@wp.pl (P.B.); 2 Faculty of Biology and Environmental Sciences, Cardinal Stefan Wyszynski University in Warsaw, Wóycickiego 1/3, 01-938 Warsaw, Poland; agczyzewska1@gmail.com

**Keywords:** microplastics, *Aiptasia*, survival, polypropylene, polyethylene

## Abstract

Microplastics are an increasingly common threat to the aquatic environment, and, due to their small size, it is easy for them to spread and enter the seas and oceans. Micro- and mesoplastic particles are often ingested by marine organisms, especially those that have the potential for successful settlement on artificial substrata, including plastic. In laboratory experiments, we tested the tendency of the sea anemone *Exaiptasia diaphana* to consume plastic fragments and fibers of various sizes and shapes; these organisms are widely distributed in coastal waters, tide pools, and coral reefs. The plastic fragments and fibers were placed either in natural food or covered with a special food in gel form, which allowed them to retain their original shape. Our studies have shown that plastic in the shape of fibers is less readily consumed than in the form of fragments. The *E. diaphana* anemones with oral discs of diameter 10–12 mm had difficulty consuming long fibers. A total of 67% of the studied animals were unable to consume fibers of 13 mm length, while those of 3 and 7 mm length were consumed by 100% of the sea anemones. We have also established that microplastics taken with food are harmful to these cnidarians: mechanical injury to the body column was caused by the ingested polypropylene microfragments, and we also observed significantly increased mortality. Injuries, combined with the possible toxicological effects of the polymers, may have been the cause of increased mortality of the *Exaiptasia diaphana*.

## Simple Summary

The small size of micro- and mesoplastics and their significant occurrence in aquatic environments leads to a high probability of continuous uptake of fragments or fibers by a number of marine organisms. We indicated that sea anemones *Exaiptasia diaphana*, widely distributed in coastal waters, tide pools, coral reefs and artificial ecosystems such as aquaria, are able to consume polypropylene fragments and polyethylene fibers. In our experiment, fibers were less readily consumed than fragments and 67% of the studied animals were unable to consume long mesofibers. The ingestion of polypropylene fragments provided with food caused mechanical injury to the body column of sea anemones and significantly increased their mortality.

## 1. Introduction

Over the past few decades, both the manufacture and use of plastics has increased quite dramatically [1,2]. Plastics are synthetic materials, with properties such as physical and chemical resistance, ease of processing and coloring, and low production costs; they are used in almost all aspects of human life. Plastics are commonly used in the construction, medical, and transportation industries, as well as in the household industry [3]. Due to their great popularity, the production of plastics is growing rapidly year-on-year, replacing previously used natural products. Unfortunately, this has consequences for the natural environment because a significant fraction of deployed plastic leaks into the environment at an uncontrolled rate [4]. One of the increasingly observed threats to the aquatic ecosystem has been microplastic (MP)—small plastic particles with a size not exceeding 5 mm [5,6]. Due to their small size, MPs are very easily spread and enter rivers, lakes, and oceans. They are most often found in coastal waters, seabed sediments, and beaches, and recent studies have shown that they have also been detected in ice of the Arctic [7] and Antarctic [4,8]. Another size class of plastic, which is found detected in natural environments is mesoplastic (MsP) usually defined as 5–25 mm [9,10]. The amount of micro- and mesoplastics and their significant occurrence in the benthic, littoral, and pelagic zones leads to a high probability of continuous uptake of fragments or fibers by a number of aquatic organisms [5,11]. MP and MsP particles are usually consumed accidentally, mainly because their size resembles the size of the prey of many marine animals [12,13]. Ingestion of MP and MsP significantly contributes to several harmful effects to organisms, such as damage to internal organs, pathological stress, reduced food intake, and reduced growth and reproduction [14].

An increasing number of reports have indicated that micro- and mesoscopic plastics have great impact on ecosystems, and this topic has not yet been fully understood and further research is still necessary and important.

Coastal oceans have become a repository for increasing amounts of plastics, leading to more and more consumption of these polymers by animals. As plastic enters the ocean, similarly to other submerged objects, it provides a habitat for a diverse community of marine organisms that are able to settle and persist on artificial substrata. Many invertebrates, including ascidians, bryozoans, and barnacles, can be found on marine litter [15,16,17]. Sea anemones, with their diverse, sessile life-history strategies, have the potential for successful settlement on artificial substrata, including plastic [18]. Teng et al. [19] observed the sea anemone *Metridium senile* attaching to a variety of seafloor litter in the Yellow Sea and suggested that accumulation of artificial substrata might be contributing to the bloom of these *sea anemones.* Sea anemones from Actiniidae and Hormathiidae families have been reported settled on rafting debris in the North Pacific Ocean [20]. The ability of sea anemones to inhabit plastic litter and polluted coasts can increase the risk of them consuming micro- and mesoplastics and suffering the consequences [21,22]. These plastic particles are ideal niches for the colonization of microbes and small organisms (biofilms also called the plastisphere communities) that can encourage predators to consume them [23,24]. The sea anemones have been reported to consume plastic particles in natural environments. For instance, the individuals of *Bunodosoma cangicum* from the intertidal zone of the Amazon coast ingested micro- and mesoplastic fibers and fragments in the range of 0.10–9.17 mm [21]. The sandy anemones *Bunodactis reynaudi* in False Bay, South Africa were able to ingest macro plastic, large (55 × 55 cm) polyethylene bags [25]. In the laboratory, anemones, e.g., *Exaiptasia pallida*, differentiated consumption depending on the type of polymer and were more likely to ingest microplastics when chemical cues of prey were present in the water [26].

There is a lack of comprehensive results on how ingested microplastics and mesoplastics affect animals, including sea anemones, and how the shape of plastic particles determines their consumption.

The aim of this study was to investigate in laboratory experiments whether plastic fragments and fibers of various sizes are consumed by the sea anemones *Exaiptasia* (=*Aiptasia*) *diaphana* and whether micro- and mesoplastics taken with food are harmful to these cnidarians. These animals have previously been shown to consume plastic microfibers and microspheres [26,27], but the organismal effect, capacities, and preferences of consuming different sizes and shapes of micro- and mesoplastic has not been analyzed. We examined how the size and shape of plastic fragments and fibers either placed in natural food or covered with a special food in gel form, which allowed the plastic fragments to retain their original shape, affects (1) the anemones’ tendency to consume it, (2) their subsequent growth, and (3) survival.

## 2. Materials and Methods

### 2.1. The Study Animal

We used sea anemones *Exaiptasia* (*=Aiptasia*) *diaphana* (Rapp 1829) syn. *Aiptasia pallida*, *Exaiptasia pallida* [28] obtained from culture aquaria at the Laboratory Center for Natural Sciences UKSW. The animals used in the laboratory experiment had previously been kept in a tank with a current flow system supplying filtered artificial seawater (salinity 33.5‰) with a controlled temperature (23 °C) and photoperiod LD12:12. The cultured *E. diaphana* were fed once a week with thawed brine shrimp and krill. These anemones mainly feed on plankton and detritus, but they also have the ability to obtain food from the photosynthesis of symbiotic algae [29]. They are characterized by their ability to spread rapidly and colonize different habitats and are considered a harmful invasive species [30]. These sea anemones are widely distributed in the marine waters of temperate and tropical zones [31]. They are most common in shallow waters, tide pools, and coral reefs but also appear in artificial habitats such as aquariums [29,32]. *Exaiptasia diaphana* individuals are easy to cultivate in the laboratory thanks to their ability to survive in a wide range of salinities and different sea water qualities. For this reason, they are often used as model organisms for scientific research [33].

### 2.2. Sea Anemones Consuming Food Contaminated by Microplastic

The laboratory experiment on the effect of plastic consumption was conducted in 2022 and lasted 14 days (until no further changes were noted). We used 60 containers with a diameter of 120 mm (20 per treatment). Each of them was filled with 400 mL of sea water with a salinity of 29‰, prepared in the laboratory based on sea salt. The containers were placed on the counter under large lamps emitting white light. One similarly sized *E. diaphana* with oral disc diameter of 10–12 mm measured in relaxed anemones (the length of the column was about 1.7 times the diameter of the oral disk) was transferred to each container (*n* = 60) using a pipette and left for 48 h to acclimate to the new environment. There were three treatments: C—control fed with frozen krill, which was thawed before feeding; F—fed with frozen krill, which was thawed and then a polyethylene (PE) fiber (fishing line 0.06 mm in diameter) not exceeding 5 mm in length (< 0.1 mg) was placed inside using micro surgical tweezers (Chirmed^®^, Rudniki, Poland) to simulate prey containing plastic.; P—fed with frozen krill, which was thawed and a polypropylene (PP) rectangular fragment, not exceeding 5 mm in length (<0.3 mg) was placed inside using micro surgical tweezers. Food was served individually using tweezers and the anemones were observed to notice whether the contaminated food was accepted or not. Then, after 24 h, we observed whether the tested organisms ingested or rejected the food with plastic given to them the day before (one portion of food—one krill, per individual). Then, approximately 5 days after feeding, the water in the containers was changed, *E. diaphana* individuals were measured again (the oral disc was measured in relaxed anemones with an accuracy of 1 mm), and growth and survival were noted.

### 2.3. Sea Anemones Consuming Micro- and Mesoplastics Covered with a Special Food

We tested the selectivity of *E. diaphana* in consuming MPs of different sizes and mesoplastic in a laboratory experiment. The selectivity experiment was divided into two treatments: F which were fed with PE fiber and P which were fed with PP rectangular fragment. Each of the 18 containers with a diameter of 70 mm was filled with 80 mL of artificial sea water with a salinity of 29‰. Then, one sea anemone of the genus *Exaiptasia* was transferred to each of them using a pipette. Individuals placed in the plastic containers were left for 24 h to acclimate to the new environment. The two types of plastic were divided into three size classes each: PE fibers (fishing line 0.06 mm in diameter) of 3 mm, 7 mm, and 13 mm (~0.06 mg, 0.13 mg, 0.25 mg, respectively) and PP rectangular fragments of 1.5 mm, 3 mm, and 5 mm (~0.15 mg, 0.3 mg, 0.5 mg, respectively). The size of plastic was selected based on literature data concerning pollution of coral reefs where fibers and fragments ~3 mm in size dominated and polyprophylene (PP) and polyethylene (PE) were the most common polymers [34,35]. Since sea anemones are “sit and wait” predators and capture a prey using cnidae that react to the presence of amino acids [36], appropriately sorted micro- and mesoplastics were briefly immersed in a special food in gel form designed for omnivorous fish (Gel Formula by Tropical) and allowed to congeal. This procedure allowed the plastic fragments and fibers to retain their original shape and be willingly taken by sea anemones. The food prepared in this way was fed to the organisms using tweezers according to individual experimental treatments (one portion of food per individual). All the anemones after catching the served plastic covered with a special food were left for 24 h to observe whether each individual was able to ingest the micro- and mesoplastic. After 24 h, we checked whether the test anemones had eaten the scraps of microplastic covered with small amounts of jelly-like food given to them the day before.

### 2.4. Statistical Analyses

Normal distribution was tested using the Shapiro–Wilk test. Normal distribution in the samples was not confirmed, and non-parametric tests were thus used: the Kruskal–Wallis test and the Mann–Whitney test to analyze the differences in percentage of sea anemones that consumed micro- and mesoplastics and their growth and survival. A significance level of α = 0.05, 0.95 confidence interval was used for statistical analysis. All statistical analyses were performed using Statistica 12 software (StatSoft, Inc., Tulsa, OK, USA).

## 3. Results

### 3.1. Changes in the Oral Disc Size of the Exaiptasia diaphana Individuals Fed with Microplastics

As a result, on the last day of measurement, the average changes in size of the individuals’ oral discs were 4 ± 1 mm independently of the treatment. Thus, the growth of the control anemones and those fed with polypropylene pieces of plastic or polyethylene fibers did not differ significantly (*p* > 0.05).

### 3.2. Survival of the Exaiptasia diaphana Individuals

**Figure 1:**
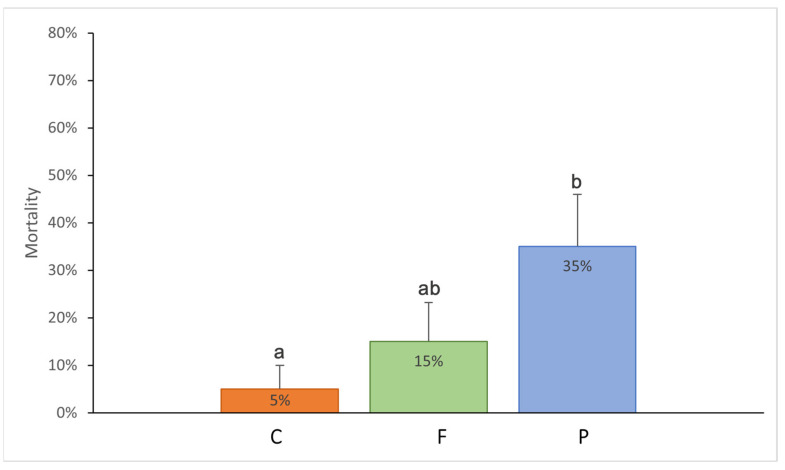
Mean mortality of the *Exaiptasia diaphana* individuals in different experimental treatments: C: control treatment, the anemones consuming krill without plastic; F: the anemones consuming krill with PE fishing line placed inside; P: with PP rectangular fragment placed inside. The error bars represent ±1 SE. Different letters indicate statistical differences at a *p* value of <0.05.

At the end of the laboratory experiment, mortality was recorded in all experimental treatments and differed significantly (Kruskal–Wallis test, H_2,60_ = 6.13, *p* = 0.047). The treatment in which *E. diaphana* individuals were fed with PP plastic had the highest percentage of dead anemones (35%), while the control treatment had the least (5%). The difference was statistically significant (Mann–Whitney test, *p* = 0.02; Figure 1).

**Figure 2:**
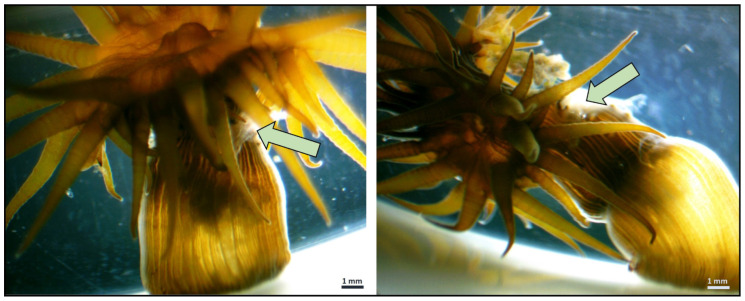
Mechanical injury to *Exaiptasia diaphana* sea anemone caused by the ingested PP rectangular fragment. The arrow indicates the injury to the body column: endo- and ectoderm are broken; mesoglea is visible.

We also observed mechanical injury (defined as a break in the body’s covering) to the sea anemone body caused by ingested PP rectangular fragments (Figure 2). However, the harmed anemones accounted for no more than 6% of all examined individuals.

### 3.3. Percentage of Sea Anemones Consuming Different Microplastics

**Figure 3:**
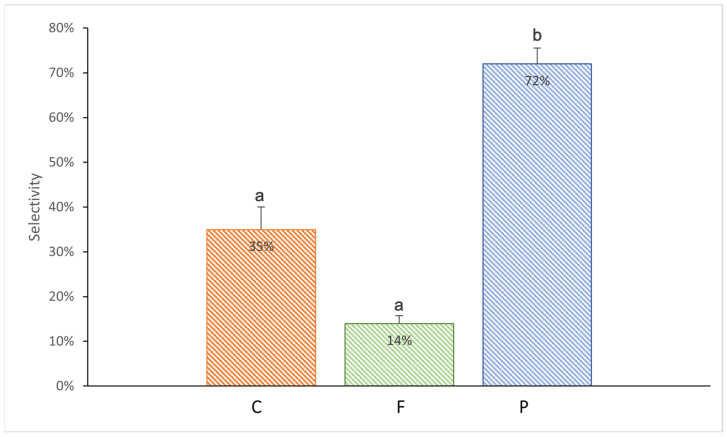
Mean selectivity (%) of the *Exaiptasia diaphana* individuals in consuming different microplastic in krill: C: the control anemones consuming krill without plastic; F: PE fishing line placed inside; P: krill with PP rectangular fragment placed inside. The error bars represent ±1 SE. Different letters indicate statistical differences at a *p* value of <0.05.

The sea anemones showed varying willingness to take microplastic with food depending on its shape (Kruskal–Wallis test, H_2,18_ = 10.29, *p* = 0.006). During the entire duration of the experiment, the PP rectangular fragments were consumed by the highest percentage of individuals (72%) (Mann–Whitney test, *p* = 0.03; Figure 3). The least readily taken food was that with PE fishing line—only 14% of sea anemones consumed it (Mann–Whitney test, *p* = 0.005 compared to the P treatment; Figure 3).

### 3.4. Selectivity in Consuming Micro- and Mesoplastics Covered with a Special Food

**Figure 4:**
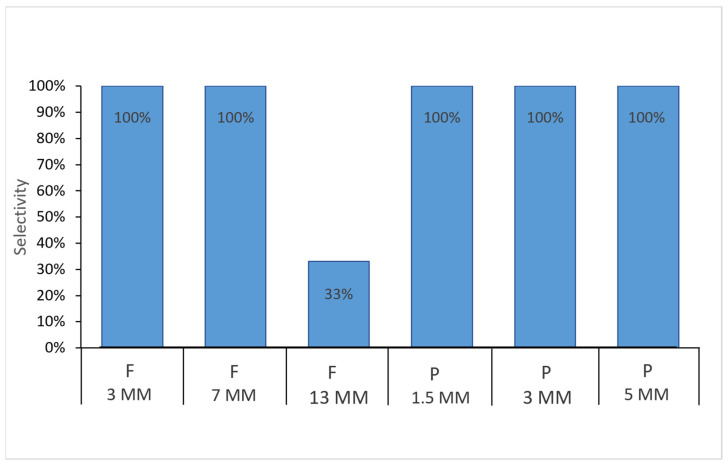
Selectivity (%) of the *Exaiptasia diaphana* individuals in consuming micro- and mesoplastic of different shape and size covered with a thin layer of special food in gel form: F: PE fishing lines in length of 3 mm, 7 mm, and 13 mm; P: PP rectangular fragments in length of 1.5 mm, 3 mm, and 5 mm.

The results of the selectivity experiment indicated that *E. diaphana* individuals can consume micro- and mesoplastics of different shapes and sizes. All sea anemones consumed the PP rectangular fragments of 1.5 mm, 3 mm, and 5 mm and PE fishing lines of 3 mm and 7 mm covered with a special food in gel form (Figure 4).

**Figure 5:**
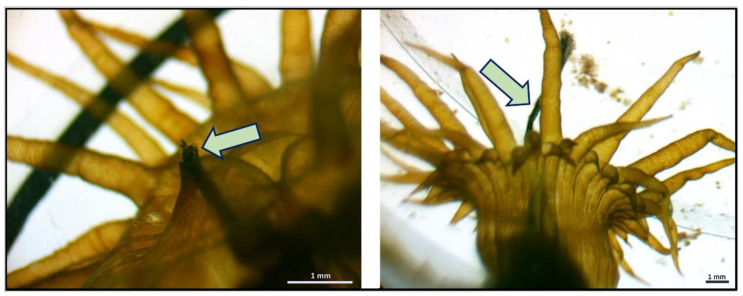
Attempts to retrieve and eventually spit out a 13-mm-long fishing line by an *Exaiptasia diaphana* sea anemone. The arrow indicates the fiber.

Interestingly, we found that the size of the plastic mattered for the fishing line. The 13 mm-long PE line was only consumed by 33% of the individuals (Figure 4). This shape of mesoplastic was taken with difficulty by the sea anemones (Figure 5).

## 4. Discussion

The *E. diaphana* sea anemones examined in this study showed differences in consumption depending on the shape of micro- and mesoplastics provided with food. The sea anemones had a greater tendency to consume fragments of PP plastic covered in food than to consume PE fishing lines fed in the same way. Similar results were obtained in a study by Gray and Weinstein [37], whose observations suggested that *Palaemonetes pugio* shrimps exposed to MPs for three hours also preferred to consume plastic fragments rather than fibers. Hence, shape has a significant impact on the choice of MP taken up by marine organisms. Fragments in the shape of granules or irregular particles may be incorrectly identified as food particles rather than fibers. For instance, planktivorous fish *Engraulis japonicus* consumed mostly fragments (86%) [38]. Moreover, the reason why marine organisms consume plastics may be due to the presence of organic compound aromas on the surface of plastics. It is surmised that plastics themselves may have substances that attract certain organisms to consume them, known as phagostimulants, which have, for example, promoted ingestion by corals and other cnidarians [26,39].

The size of plastic particles is also important in the preference for uptake, as it plays a significant role in the ecological context—mainly because it is a key factor affecting the particles’ interaction with biota. Our studies showed that the *E. diaphana* sea anemones could take microplastics of varying lengths. Sea anemones have a great capacity to consume relatively large prey. Nevertheless, mesoplastic, such as a 13-mm-long fishing line, posed a problem for them due to its size. Smaller marine organisms may find it difficult to consume larger-sized mesoplastics, even though they are believed to be easier to detect. While there is a growing number of studies on micro- and mesoplastic preferences, it is important to consider that different species of organisms may consume different sizes. For instance, Jâms et al. [40] estimated that a zooplankton animal 13.5 mm in length is able to ingest a piece of plastic 0.86 mm long. In eastern oysters (*Crassostrea virginica*), accumulation of microplastics is size-dependent, with tendencies for accumulation toward longer fibers > 250 μm and smaller fragments < 500 μm [41]. Freshwater *dragonfly Anax imperator* larvae can ingest mesoplastic fibers 8 mm long and, as a result of mechanical comminution, turn them into microplastics 0.5–3.5 mm in size [42]. Our studies indicated that the *E. diaphana* sea anemones with oral discs of diameter 4–5 mm can successfully consume rectangular plastic fragments up to 5 mm and fibers up to 7 mm.

Coral reefs, which are already exposed to many stressors associated with human activities, such as increased water temperatures, ocean acidification resulting from global climate change, and overfishing, are particularly sensitive to the impact of microplastics. Such impacts may have long-term effects on tropical ecosystems and associated organisms. With increasing reports of microplastic contamination of coral reefs, our experiments aimed to investigate the potential impact of two types of polymers—polypropylene (PP) and polyethylene (PE)—on *E. diaphana* sea anemones. Both polymers are among the most commonly used plastics in the world and have been reported as dominant on coral reefs and coastal zones [34,35,43]. When fragments of plastic are ingested by aquatic organisms, there are usually a number of negative effects, including decreased ability to consume food; pathological stress; damage to internal organs; and reduced survival, growth, and reproduction [14]. The results of our studies indicated increased mortality of sea anemones that consumed polypropylene (PP) rectangular fragments with their food. We observed that plastic fragments could cause direct injuries to the endoderm and even complete puncture of the body wall and damage to the ectoderm. Murphy and Quinn [44] found that exposure to PE microplastics caused morphological impairment of the freshwater cnidarian *Hydra attenuate*, however, this effect was non-lethal. Increased mortality due to microplastic-contaminated food has been widely reported for marine and freshwater invertebrates, such as crustaceans and mussels [45,46,47]. Leung and Chan [48] indicated that polystyrene microplastic exposure increased mortality and reduced the rate of posterior segment regeneration in polychaete *Perinereis aibuhitensis.* The polyp mortality was reported for corals *Astrangia poculata* that ingested polyethylene microbeads. The reason was probably microplastic-assisted delivery of *Escherichia coli* and subsequent infection of coral tissues [49]; microplastics have been reported as substrates for microorganism colonization and a vector for pathogen spreading [50,51]. Injuries, combined with the toxicological effects of polymers and possible bacterial infection, may have been the cause of the increased mortality of the *Exaiptasia diaphana* observed in our studies. Despite the increasing number of studies on the harmfulness of micro- and mesoplastics to animals, it is still not known exactly how plastic may affect different groups of organisms. Our research indicated that even such organisms as cnidarians, with a high capacity for regeneration, suffer from this type of pollution. Further research to understand the negative effects of various plastic polymers, comparisons of populations from environments with different degrees of pollution, and the extent of possible plastic-induced damage seems necessary.

## 5. Conclusions

The size of plastic particles is important in the preference for uptake by animals, as it plays a significant role in the ecological context—mainly because it is a key factor affecting the plastic interaction with biota. Due to their small size, microplastics (MPs; <5 mm) are very easily spread and enter all parts of marine, freshwater, and land environments. Another size class of plastic detected in natural environments is mesoplastic (MsP; 5–25 mm). The amount of mesoplastics as well as microplastics and their significant occurrence in environments leads to a high probability of continuous uptake of fragments or fibers by a number of aquatic organisms. Sea anemones have a great capacity to consume relatively large prey. Our studies showed that the *Exaiptasia diaphana* sea anemones with oral discs of diameter 4–5 mm were able to consume rectangular plastic fragments up to 5 mm and fibers up to 7 mm. Mesoplastic, such as a 13-mm-long fishing line, posed a problem for them due to its shape and size. We indicated negative effect of the plastic ingestion—increased mortality of sea anemones that consumed polypropylene (PP) rectangular fragments with their food. These fragments could cause direct injuries to the endoderm and even lead to complete puncture of the body wall and damage to the ectoderm. We assume that the toxicological effects of polymers, injuries, and possible bacterial infection could have been the cause of the *Exaiptasia diaphana* increased mortality.

## Data Availability

The data that support the findings of this study are available on request from the corresponding author.
